# Environmental Enrichment for Rats and Mice Housed in Laboratories: A Metareview

**DOI:** 10.3390/ani12040414

**Published:** 2022-02-09

**Authors:** Anna S. Ratuski, Daniel M. Weary

**Affiliations:** Animal Welfare Program, Faculty of Land and Food Systems, University of British Columbia, Vancouver, BC V6T 1Z4, Canada

**Keywords:** refinement, laboratory animals, rodent, animal welfare, environmental complexity

## Abstract

**Simple Summary:**

Environmental enrichment has been widely studied with laboratory rodents, but there is no consensus regarding what counts as enrichment or what it should achieve. Inconsistent use of the term “enrichment” creates challenges in drawing conclusions about the quality of an environment. We conducted a metareview to better understand the definitions and goals of enrichment, perceived risks or requirements of enrichment, and what forms of enrichment have previously been endorsed for use with rodents housed in laboratories. This may help researchers and animal care staff to better define their chosen approach and intended outcomes when providing environmental enrichment.

**Abstract:**

Environmental enrichment has been widely studied in rodents, but there is no consensus on what enrichment should look like or what it should achieve. Inconsistent use of the term “enrichment” creates challenges in drawing conclusions about the quality of an environment, which may slow housing improvements for laboratory animals. Many review articles have addressed environmental enrichment for laboratory rats and mice (*Rattus norvegicus* and *Mus musculus*). We conducted a metareview of 29 review articles to assess how enrichment has been defined and what are commonly described as its goals or requirements. Recommendations from each article were summarised to illustrate the conditions generally considered suitable for laboratory rodents. While there is no consensus on alternative terminology, many articles acknowledged that the blanket use of the terms “enriched” and “enrichment” should be avoided. Environmental enrichment was most often conceptualised as a method to increase natural behaviour and improve animal welfare. Authors also commonly outlined perceived risks and requirements of environmental enrichment. We discuss these perceptions, make suggestions for future research, and advocate for the adoption of more specific and value-neutral terminology.

## 1. Introduction

There are many incongruencies between the natural adaptations of mice (*Mus musculus*) and rats (*Rattus norvegicus*) and the laboratory conditions in which they are typically housed (for mice, see [1,2]; for rats, see [3,4]). There has been some speculation on whether laboratory rodents have the same needs as their wild counterparts, with some arguing that laboratory rodents are adapted to life in cages (e.g., [5,6,7]). While it is reasonable to assume some differences in behaviour after generations of captive breeding, there is little evidence that domestication results in a reduced behavioural repertoire. Rather, domestication and captive environments tend to alter the frequency of behavioural expressions and the strength or nature of stimuli required to elicit them [8]. When given access to a burrowing substrate, for example, mice and rats in laboratories readily perform this behaviour [9,10,11,12]. Rather than accommodating a full repertoire of behaviours, housing for rodents used in research has largely focused upon standardisation and convenience for humans [13].

While some improvements to housing have been made (e.g., the shift from suspended wire cages to solid-bottom cages with bedding [14]), there is mounting evidence that current practices negatively impact welfare. Laboratory cages restrict the expression of behaviours such as burrowing, foraging, exploration, climbing, and more complex social interactions. Captive environments that prevent natural behaviours can cause frustration, stress, depression, and behaviours indicative of poor welfare [15,16]. For rodents, conventional laboratory cages (which we define here as polycarbonate cages with bedding and possibly nesting material and/or a shelter, with food pellets provided in a metal food hopper) can lead to the development of stereotypic behaviours [17,18,19], weakened immune responses [20,21], increased acute hormonal responses to stressors [22], and behaviours indicative of anxiety [23,24] and pain [25]. Individually ventilated cages are also increasingly used, offering increased biosecurity but impairing animal thermoregulation [26].

Early versions of guidelines for laboratory animal husbandry have typically excluded environmental enrichment for rodents (e.g., [27]). This is changing with time, with some guidelines providing minimally acceptable enrichment components [28,29] and others encouraging enrichment more generally [14]. In recent years, there has been a push to improve the lives of animals used in research (e.g., [30,31]). To use animals ethically in research, some authors have suggested that we must provide them with environments that promote a “good life” [32] or maximise the potential for an animal’s needs to be fulfilled [33]. Beyond ethical arguments, there are pragmatic concerns relating to the effect of poor animal welfare on the quality of scientific results [34,35]. As such, the use of environmental enrichment has been promoted for animals housed in laboratories.

### What Is Enrichment for Rodents?

Definitions and applications of environmental enrichment tend to vary widely between studies. Indeed, the environmental enrichment literature has been criticised for inconsistent usage of terms and lack of precise definitions (e.g., [36,37]). Some studies (all from roughly the last decade) refer to the addition of nesting material (to an otherwise barren cage) as enriched (e.g., [23,38,39]), although in other studies a cage with nesting material is sometimes used as a condition representing no enrichment (e.g., [40,41]). Other studies refer to cages containing nesting material in addition to other components as their conventional or non-enriched condition (e.g., [42,43]), and many studies do not describe their conventional housing conditions at all [21], making direct comparisons between studies difficult. Because the term is variously applied, an otherwise barren cage altered in any way might be labelled as “enrichment.” As one example of such practices, a shoebox cage for mice modified to have one indented corner has recently been marketed as enriched [44]. Such usage of the term dilutes its meaning, promotes continued provision of sub-optimal environments, and creates challenges in drawing conclusions about the effects of enrichment.

Environmental enrichment has been widely studied with rodents. Approximately 70% of enrichment studies from 1985 to 2004 focused on laboratory animals, especially rodents. Publications on this topic appeared in the literature with increasing frequency from 1999 onward, related to growing interest from neuroscientists [45], and this trend has continued to date [46]. A PubMed search for “environmental enrichment” and “rats” or “mice” identified 662 publications from 2020 alone. This may be related to increasing use of the ARRIVE guidelines [47], which recommend disclosing information on housing and husbandry in publications (however, a recent revision has moved environmental enrichment into the category of recommended rather than essential reporting [48]). Despite increasing information surrounding enrichment use with rodents, varied definitions and applications of enrichment contribute to difficulty in drawing conclusions. In neuroscience research, enrichment is often conceptualised and applied as a research paradigm assessing the effects of environmental complexity or novelty on disease progression, brain development, and brain function. Outside of neuroscience research, enrichment is often conceptualised as an application intended to enhance animal welfare, with some scholars labelling all environmental components as “enrichment”, and others reserving the term only for the provision of components that surpass minimum environmental requirements. These different approaches to the conceptualisation of enrichment have likely contributed to variation in its perceived effects.

With growing interest in the topic, it is important to clarify how enrichment is conceptualised and applied. Many review articles have summarised and discussed environmental enrichment, providing commentary on both theoretical and practical aspects. The present article provides a metareview of these review articles, summarising how enrichment has been defined, what are described as its goals, and what are perceived as risks or requirements of enrichment. Recommendations from each article are also summarised to illustrate the range of conditions considered appropriate for laboratory rodents. We conclude with a general discussion of how to move forward with the application and study of environmental enrichment for rats and mice.

## 2. Methods

We emulated the approach of a systematic review such that our search strategy and inclusion criteria were comprehensive and could be replicated. However, our aim was to clarify key concepts and definitions from the literature rather than to perform a meta-analysis of primary research findings; therefore, our methodology aligns with that of a scoping review rather than a systematic review [49]. Given that we focused only on review articles in our search, we have used the term “metareview”.

### 2.1. Search Strategy

A search was performed on three academic databases (Web of Science Core Collection, PubMed, and CAB Direct) on 1 March 2021. The following search terms were used on Web of Science: ((rodent OR rat OR mice) AND (“environmental enrichment” OR enrich* OR caging OR cage* OR housing OR environment)). We also specified document type (review articles). This search yielded 65 publications. The same search terms were used on CAB Direct; results were further refined by available topics: reviews, laboratory animals, animal research, animal husbandry, animal models, behaviour, animal behaviour, animal welfare, or animal housing; this search yielded 185 publications. On PubMed, the following search terms were used: ((rat) OR (mice) OR (rodent)) AND (animal welfare) AND ((“environmental enrichment”) OR (enrich* adj1 (environment OR cage* OR caging OR housing))); this yielded 98 publications. All references were loaded into Covidence systematic review software and duplicates were removed. Titles and abstracts were screened using our inclusion criteria described in the following section. Full text screening and data extraction were performed independently by two reviewers. All articles that met the inclusion criteria had their reference lists screened for potential additional articles.

### 2.2. Inclusion Criteria

No publication date limits were imposed. Primary research articles and books were excluded. Only articles written in English were included. Articles had to be focused on “environmental enrichment” (or any described alternate term such as environmental complexity) for laboratory rats or mice; articles discussing a variety of species used in laboratories were acceptable if there was commentary dedicated to rats or mice. We did not seek out reviews on specific forms of environmental enrichment (e.g., social housing). Included articles had to discuss the use of environmental enrichment as a method to refine housing or husbandry practices; reviews on use of environmental enrichment purely as an experimental paradigm or therapeutic intervention (i.e., for disease models) were excluded. Articles did not need to be comprehensive or systematic reviews. As such, many articles addressed specific aspects of enrichment, such as its “unintended outcomes” [50], provision of foraging opportunities [51,52], and effects on variability of research outcomes [46,53,54]. Reported summaries of recommended enrichment strategies should be interpreted with this in mind, as failure to discuss a specific type of enrichment within one context does not mean that it would be excluded in another.

## 3. Results and Discussion

**Figure 1:**
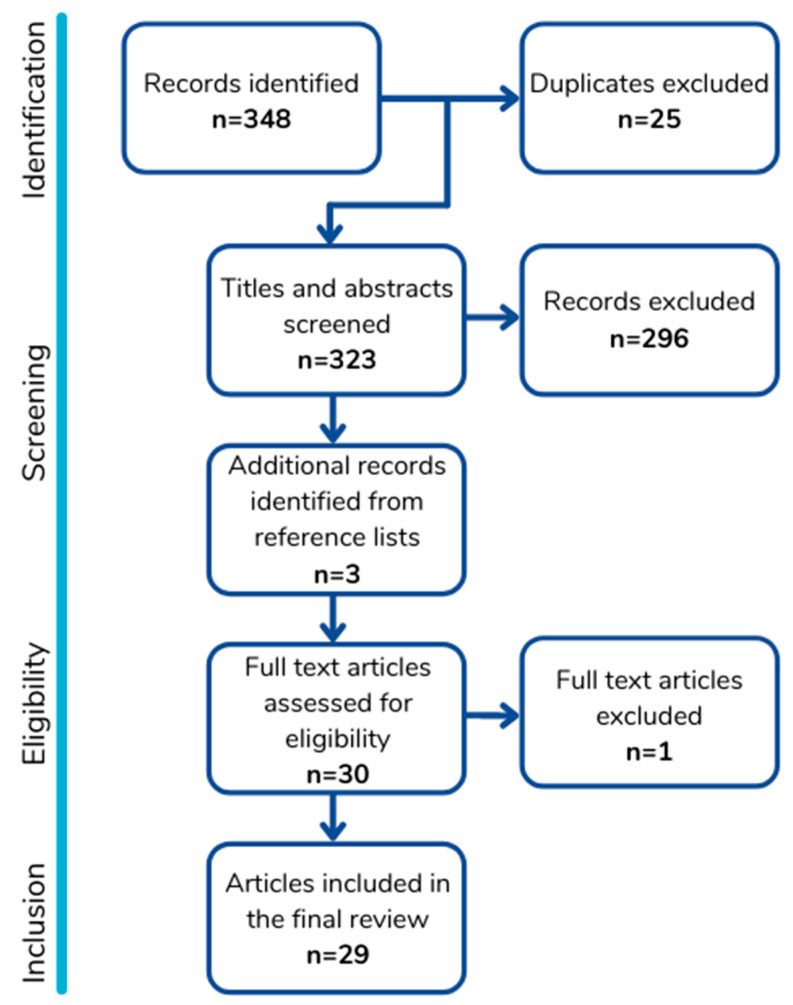
Diagram showing our process of identifying and screening articles for eligibility and inclusion, resulting in the 29 articles included in this metareview.

In total, 29 articles were included (Figure 1). One article was excluded at the full-text screening stage because it was focused on the use of enrichment as an experimental paradigm only and did not include discussion of general husbandry practices. Articles were published over a 30-year span (from 1991 to 2021). Data are summarised in Table 1.

In the sections that follow, we outline the main findings from Table 1, critically discussing the prevalent themes. Definitions and goals of enrichment are discussed together within the same section. We then discuss enrichment components recommended in these reviews. Lastly, the risks or requirements of enrichment are discussed. We end with a general discussion concerning enrichment for rodents, as well as conclusions and recommendations for future work.

### 3.1. Environmental Enrichment Definitions and Goals

Most articles acknowledged that the blanket use of the terms “enriched” and “enrichment” should be avoided, but alternative terminology was not agreed upon. Within the articles reviewed, six alternatives were suggested to replace the term “environmental enrichment”. Olsson and Dahlborn [37] (p. 246) suggested “changes to or modifications of the environment or housing conditions,” to avoid implying that all modifications are positive for animals. Similarly, Benefiel et al. [63] (p. 96) proposed the term “housing supplementation” as an alternative to enrichment, again avoiding implications that all changes benefit the animals. Baumans and Van Loo [69] (p. 26) used the term “environmental refinement” to indicate the ongoing process of providing more than just the basic needs of animals, highlighting that the term “enrichment” is sometimes taken to imply that animals are provided with luxuries. Pritchett-Corning [74] (p. 239) used the descriptive (rather than evaluative) term “environmental complexity”, explaining that this ranges from barren to naturalistic conditions, and that varying categories of environmental enrichment (standard, superenriched, and semi-naturalistic) exist within this range. Sørensen et al. [61] used the terms enrichment and complexity interchangeably. Würbel and Garner [54] (p. 3) differentiated between possible enrichment outcomes, using the term “beneficial enrichment” for enrichment that is biologically relevant and improves animal welfare, and referring to enrichment attempts that are not biologically relevant or potentially harmful as “pseudoenrichment.” “Superenrichment” was defined separately from environmental enrichment in two articles, without consensus [64,74]. Our own view is that despite the shortcomings of the term, “environmental enrichment” is so engrained in the literature that it is unlikely that any alternative will soon replace it. Going forward, we suggest that the term “enriched” should be accompanied by an explicit description, including what a chosen strategy is aiming to achieve and criteria for success.

Overall, definitions were largely focused on what enrichment should achieve; few described what enrichment should resemble. Some mentioned the theme of increasing environmental complexity or naturalness, but were not prescriptive as to what this should entail. Three articles did not provide a definition of environmental enrichment.

The most common elements of definitions were that enrichment changes the environment to improve animal welfare, facilitate natural or species-typical behaviours, and increase engagement or stimulation. Improving animal welfare and increasing natural behaviour were also the most commonly mentioned goals of enrichment (mentioned in 23 and 20 articles, respectively). These are different but potentially overlapping aims, depending on how animal welfare is conceived. Much like environmental enrichment, animal welfare does not have one universally agreed upon definition or framework. For example, Fraser et al. [75] consider welfare as three overlapping spheres: natural living, biological functioning, and affective states (emotions or feelings), but others have emphasised biological functioning [76] or fulfilment of an animal’s evolutionary nature [77]. Dawkins [78] has suggested that animal welfare is the combination of health and what animals want, precluding naturalness as inherently important to welfare, and rather favouring animal preferences and motivation. Other attributes, such as the ability for animals to exercise agency, have also gained traction in recent years [79,80]. Thus, improving welfare can mean different things to different people.

Several authors cited a definition describing enrichment as an improvement in biological functioning as a result of environmental changes [36], indicating that health and biological functioning are central to their idea of welfare. Reviewed articles mentioned a variety of goals that fit under the umbrella of animal welfare and may be indicative of different frameworks or priorities: improving health or biological functioning (eight articles), improving psychological well-being (eight articles), increasing ability to cope with stressors (four articles), providing choice or control (three articles), and reducing boredom (two articles). Other definitions specified that enrichment is a method to decrease abnormal or stereotypic behaviours, or that enrichment provides animals with increased coping abilities or cognitive opportunities.

According to certain conceptions of welfare, naturalness, or the ability to express natural behaviours is an inherent concern, but for others the expression of natural behaviour may only be of value if it contributes to improving other components of animal welfare such as affective states or biological functioning (discussed in [81]). For example, Bracke and Hopster proposed the following instrumental conception of natural behaviour as “behaviour that animals tend to perform under natural conditions, because it is pleasurable and promotes biological functioning” [15] (p. 80). Newberry [36] suggested that the functionality and adaptiveness of a behaviour should be emphasised, while Mench [82] suggested that we should focus on identifying the consequences of a behaviour. If more instrumental conceptions are adopted, then understanding how specific behaviours contribute to welfare is important. However, at least for some, living a natural life may be viewed as inherent to good welfare [83].

In captive settings at least, some “unnatural” behaviours may promote positive affective states or biological functioning, while some more natural behaviours may have the opposite effect. As one example, neuroscientists trained rats to drive a car (to measure the effects of environmental enrichment on learning abilities), and noted that the training process seemed to serve as a form of enrichment for all rats [84]. Some articles stated more specifically that a goal of enrichment is to increase positive natural behaviours [7,37,58]. One article suggested that enrichment should strive to keep animals docile or tame rather than encouraging behaviours typical of wild animals given that some of these behaviours, such as avoidance of humans or reactivity to handling, are seen as unsuitable for life in the laboratory [56]. We suggest that enrichment strategies that increase behavioural diversity (see [85]) or provide increased agency (i.e., the ability to exert control over their environment or make choices) will be instrumentally beneficial to the welfare of laboratory animals regardless of their perceived naturalness. We also recommend that authors be specific when describing which behaviours they are attempting to elicit (or supress) and why they believe this to be important for the animal.

Articles often specified that enrichment should decrease stereotypic or undesirable behaviours (13 articles), raising the question of whether enrichment is seen as more of a preventative tool or a treatment for these behaviours. Considering the reduction of stereotypic behaviour as a key goal of enrichment may lead some to conclude that environmental conditions are adequate when stereotypic behaviours are absent or rare (e.g., [7,56,63]), failing to recognise that a lack of abnormal behaviours is not evidence of good welfare [2,86]. Providing an environment that reduces the development of stereotypies is likely to be good for welfare, but once these behaviours develop, they can persist if the enrichment does not address the underlying motivation [87].

### 3.2. Commonly Recommended Rodent Enrichment Strategies

Some earlier authors were sceptical regarding implementation of enrichment, promoting a more cautious approach to even basic cage modifications (e.g., [7,56,63]); however, more supportive views also existed in earlier years (e.g., [13,55,57]), so it is unclear if perceptions have changed consistently over time. That said, more recent publications generally support the provision of more extensive environmental enrichment for laboratory rodents [46,73,74]; some more basic recommendations, like solid cage floors [55,57], were present only in earlier reviews (likely because wire flooring is no longer common). Three articles made no overall recommendations due to the lack of consensus or the perceived need for more research. Some authors may have excluded some more popular husbandry strategies (such as social housing) from their recommendations because they considered these to be a different type of husbandry component (i.e., social rather than environmental), or a standard practice rather than an improvement upon current conditions.

For rats, social housing was recommended in 17 articles, followed by larger/higher cages (10 articles), nesting material (nine articles), shelters/nest boxes (11 articles), and opportunities to forage (seven articles) or gnaw (six articles). Less commonly recommended were soft or deep bedding (four articles), digging opportunities (two articles), solid flooring or a shelf in the cage (three articles), regular introduction of novelty (one article), auditory enrichment (one article), appropriate lighting (one article), and hard pelleted food (one article). One article recommended against increasing cage sizes, and another recommended against “superenriched” conditions.

For mice, nesting material was most often recommended (18 articles), followed by social housing (14 articles), shelters/nest boxes (12 articles), foraging opportunities (seven articles), and larger cages (six articles). Other recommendations included gnawing opportunities (four articles), appropriate lighting (two articles), burrowing (one article), climbing opportunities (one article), positive reinforcement training (one article), and auditory enrichment (one article). Running wheels were only conditionally recommended (one article), as were several other forms of enrichment due to concerns about the potential for aggression or individual differences between animals. One article recommended against marbles (as an example of pseudoenrichment), one recommended against larger cages, and one argued against the use of “superenriched” conditions. Support for social housing and larger cages (compared to the current conventional cage sizes) was noted more often for rats than for mice; this may be related to the relatively more restrictive dimensions of existing rat cages, or concerns about mouse aggression in larger cages. Several recommendations were made conditionally; for example, many forms of enrichment were recommended for female mice but not for males due to sex-specific differences in outcomes.

Different authors may have conceptualised categories of enrichment differently. For example, some authors specified that rodents should be provided with shelters, while others used the term nest box, but often too little detail was provided to determine if the terms were being used synonymously. The common practice of providing rats with a tunnel or section of PVC pipe may not be in line with recommendations if authors consider nest boxes and/or shelters to be structurally different from open-ended tunnels—one article specifically recommended against the use of tunnels as shelters (likely because rats do not prefer open-ended tunnels [88]). Such discrepancies highlight the need for specificity in enrichment descriptions.

Discriminating between different applications of enrichment (i.e., a housing refinement vs. an experimental treatment for biomedical or neuroscience research) is important given that there is some discrepancy between what is commonly applied in experiments and what is considered functionally appropriate for rodents. “Toys” (typically used when referring to a wide range of inanimate objects in a variety of shapes and colours) are commonly used in enrichment research [46,68]. Some studies have included billiard balls [89] or checkers tiles and ping-pong balls [90] in their “enriched” conditions. It is worth noting that toys were not generally recommended for rodents in the articles we reviewed, and have been criticised as functionally irrelevant [36]. Another term, manipulanda (defined as “any objects that can be altered by an animal or encourage it to engage in fine motor movements” [64] (p. 151)), is more commonly used in the enrichment literature and tends to include examples of functional items which have been recommended for rodents, such as gnawing devices.

Some enrichment methods are well studied and were frequently endorsed in review articles. We suggest that several of these more frequently endorsed elements (i.e., social housing, nesting material, shelters, foraging opportunities) are now sufficiently established that they should be regarded as basic to good rodent housing conditions rather than “enriched”, or that systems that fail to include these features be considered impoverished. Others have suggested that conventional housing should be more formally recognised as a stressful laboratory procedure [21]. Such framing may help motivate change in laboratories that until now have been unable or unwilling to include these features.

### 3.3. Risks or Requirements of Enrichment

Most articles in Table 1 described perceived barriers to implementing enrichment, which we have summarised as anything identified as a risk (i.e., a potentially negative outcome) or a requirement that must be met for enrichment to be implemented. The most often cited risks and requirements were (followed by the number of articles citing this theme): enrichment programs must account for biological differences (e.g., species, strain, age, or sex; 16); financial constraints (14); practical issues (e.g., increased labour; 14); risks of increased variability (14); risks of altered research outcomes (13); enrichment must have empirically proven benefits (12); and risks to animal health or safety (12). Each of these risks or requirements are discussed in more detail in the sections that follow.

#### 3.3.1. Enrichment Programs Must Account for Biological Differences

Most articles stated that enrichment must be assessed for a range of factors such as species, age, strain, and sex to ensure that it is biologically appropriate. Indeed, reactions to enrichment may vary according to genetic strain [91] or sex (especially for mice, discussed in Section 3.3.7). Related to this notion, some articles promoted assessment of enrichment on a case-by-case basis instead of making broad recommendations [7,50,71]. While this approach might cater to biological or individual differences, it would also increase the workload related to enrichment, which was commonly cited as something to avoid. Another risk of a case-by-case approach is that the adoption of any enrichment could then be considered inappropriate by default until its effectiveness has been researched with the strain, sex, disease, etc. of interest. As outlined above, we suggest that widely studied housing features could be universally applied as the default option unless there is evidence to justify contraindication in specific instances. Minimum housing guidelines have proven to be important in the adoption of basic enrichment for rodents, and some technicians in the UK have suggested that more specific regulations could help to overcome barriers [92].

#### 3.3.2. Financial Constraints

The need for enrichment programs to be inexpensive or to make use of existing materials was emphasised in several articles. One article suggested that it would be unfair for facilities to bear the burden of investing in new housing infrastructure [65], but it is unclear who should bear this burden instead. Another article argued that funds should be made available to establish more complex housing for lab animals, but did not specify from whom [46]. In Canada, guidelines state that the convenience or cost of enrichment (either financially or in terms of labour) should not be the deciding factor regarding whether to provide enrichment [93], but a Canadian survey found that the majority of researchers considered financial costs in animal welfare decisions [94]. This gap suggests that more specific guidance is needed by regulators to reduce the risk that refinements are dismissed because of financial concerns.

In animal agriculture, the notion that improvements in animal welfare must be balanced against economic costs is common [95]. Improved welfare on farms can sometimes result in increased productivity (e.g., through improved product quality or decreased mortality [96]). Similar opportunities may be possible in laboratory animal research if improved welfare also improves the quality (e.g., reproducibility or validity) of research findings, as suggested in several articles [46,53,54,70,72].

#### 3.3.3. Practical Issues

Most articles raised concerns about the practicality of enrichment. This may be in contention with regulatory sentiments that “decisions to implement enrichment strategies should not be based on convenience where the result is to the detriment of animal welfare” [93], (p. 20). Concerns about practicality are not unique to rodents; for example, Stevens et al. [97] acknowledged the commonly raised practical concerns surrounding the implementation of enrichment in zebrafish facilities, such as increased costs or labour. Lack of time is one of the most often reported barriers to enrichment [98]. However, other work has shown that staff generally want to implement more enrichment for animals in their care, and personnel who reported less enrichment use, or the desire to provide more enrichment to their animals, also reported higher rates of burnout [99]. Animal care staff in one study reported a slight increase in workload when shelters and nesting material were introduced in their animal units, but their overall impression of the enrichment was positive [100]. Lack of time has also been identified as a major barrier to the adoption of non-aversive handling methods for laboratory mice [101] and refined euthanasia methods [102]. Likewise, in the zoo community, technicians often feel constrained by lack of time [103] or lack of institutional support rather than lack of personal motivation to implement enrichment [104]. Despite being viewed by zoo professionals as an essential husbandry practice, enrichment is often treated as a luxury due to practical barriers to its implementation [104].

Practical issues such as lack of time may require creative solutions. For example, one university implemented a program to reduce time spent on cage cleaning, streamlining the process and freeing up approximately 35 min/d of technician time that could be spent elsewhere [105]. Similar initiatives across animal research facilities may enable staff to spend more time engaging in enrichment activities. Opportunities to provide enrichment may have additional value in the form of increased job satisfaction [106] and reduced compassion fatigue for personnel working with laboratory animals.

#### 3.3.4. Risk of Increased Variability

The risk of increased variability was identified in approximately half of the reviewed articles. Increasing variability was often seen to conflict with the goal of Reduction (assuming that research under more complex environmental conditions necessitates larger sample sizes). Several articles in Table 1 suggested that the risk of increased variation was low. One article concluded that enrichment does not generally increase variation in results, although this can depend on factors such as strain, experimental parameters, and enrichment provided [53]. Another concluded that enrichment can be used without increasing within-experiment variation so long as it is species-appropriate and does not act as a stressor (otherwise variation may increase) [70]. A systematic review and meta-analysis of 281 rodent enrichment studies concluded that complex housing does not make results any more variable in comparison to conventional laboratory housing [46]. Another recent systematic review of rodents induced with stress-sensitive diseases found no effect of housing conditions on variation [21].

A series of empirical studies have largely failed to show an increase in variability resulting from enrichment. In a study using three different housing conditions, Augustsson et al. [107] found that genetic strain of the mouse had the biggest impact on variation in light–dark box behaviour, body weight, food intake, and water intake; housing conditions had minimal impact on variation. In a multi-laboratory study where enrichment conditions and age were used to systematically introduce variation, differences between laboratories were the largest source of variation for nearly every behavioural measure [108]. In another large-scale study with two strains of mice, André et al. [109] found that nesting material and shelter did not increase variability in a broad range of physiological outcomes, concluding that experimental cohort was more influential than enrichment. Mieske et al. [110] housed mice in larger cages with several connected elevated levels and concluded that the variability of their resting metabolic rate was comparable to variability found in studies using conventional housing systems. Bailoo et al. [111] found no consistent relationship between enrichment and variation for any outcome (across a range of outcomes such as anxiety, endocrine responses, growth, and brain function), even with the use of larger and more complex environments. Indeed, deliberately introducing and embracing variability within a study might help to make results more replicable and generalisable [112,113].

#### 3.3.5. Risk of Altered Research Outcomes

Several review articles focused on how enrichment might affect research outcomes [46,50,53,54,70]. However, it is important to note that results can be impacted by all environmental conditions, known or unknown; responses to status quo conditions should not be considered normal without evidence to support this assessment. Indeed, conventional housing is associated with a host of abnormal responses. For example, David et al. [26] found that mice housed in individually ventilated cages without shelters showed histological signs of chronic cold stress and altered experimental results (as measured by adrenal weights, tumour growth, and adipose tissue). Other aspects such as lighting, noise, smells, handling of animals, and other minor environmental changes can impact the animals involved in research [114].

Standardised conventional housing should not be considered to represent ideal experimental conditions given the low repeatability and translatability of preclinical animal research [115,116,117,118]. The idea that standardised housing contributes to reproducibility has been criticised (the standardisation fallacy; [119,120]). Crabbe et al. [121] assessed multiple strains of mice in three different laboratories using standardised husbandry and experimental protocols, and found effects of the laboratory in six of eight outcomes and interactions between laboratory and genotype for five outcomes. Thus, even standardised conditions can result in artefacts in the data or systematic overestimation of the effect of strain on experimental outcomes, when outcomes are in fact the result of an interaction between strain and laboratory-specific environmental conditions [17,108]. True standardisation of all environmental variables across laboratories is impossible, resulting in variation regardless of enrichment protocols; results may only be replicable if they are generalisable to a range of laboratory conditions [70,73,122,123].

Interpretations of experimental outcomes may differ depending on how housing conditions are framed. For example, we could reframe the shoebox cage as a treatment in which we are measuring the effects of persistent stressors related to an impoverished environment [124]. As described by Contreras and Rollin [33], (p. 21): “One must understand the normal behaviours, environment, and physiologic adaptations that the subject utilises to survive and thrive in the subject’s environment. If one does not appreciate normal, one cannot recognise abnormal.” Studies generally fail to acknowledge that the effects of enrichment are typically measured in comparison to animals that are predisposed to exhibit enhanced symptoms of disease [21] as a result of heightened stress in conventional cages [125] or single housing [68,126]. Enrichment is recognised as reducing symptoms for a wide range of rodent disease models, and is therefore recommended as a therapeutic treatment for human populations (e.g., [127,128]). However, even modest increases in enrichment can protect against disease in animal models; several articles have questioned the external validity and translatability of preclinical animal research using conventional housing, suggesting that “control” conditions in preclinical research should be “enriched” to improve research quality [46,129,130,131].

#### 3.3.6. Enrichment Programs Must Have Empirically Proven Benefits

Most articles specified that enrichment must have benefits proven through empirical research, with some suggesting that professional judgments are insufficient [56]. Additional conditions were related to the quantity and type of evidence required to make changes; many articles called for the study of enrichment with the measurement of both physiological and behavioural outcomes. Such data has value, but these suggestions raise important questions, such as how much evidence is required to support changes in practice, and who should be responsible for generating this? Are those who propose welfare initiatives also expected to scientifically prove benefits [55], or should the burden fall upon researchers to show that existing housing methods are appropriate? Until these criteria are more transparently outlined by policymakers, open calls for “more research” are likely to delay implementation of refinements that have been studied [59].

Even when evidence is available, it may not be considered sufficiently compelling to motivate changes in practice. For example, some of the reviewed articles suggested that data on animal preferences should not be considered sufficient to draw conclusions regarding enrichment, arguing that animals can prefer options that are harmful (illustrated through examples of rodents provided access to unbalanced diets or addictive substances, e.g., [7]). The need for other types of evidence, such as measures of motivational strength or physiological outcomes, was emphasised by several authors [37,55,63,64]. While other measures are important to form a complete assessment of animal welfare, we suggest that preference results are useful so long as care is taken to consider the influence of current environmental conditions, previous experiences, the choices offered, and the testing methods used [82,132].

Researchers sometimes call for further evidence even in cases where evidence exists. For example, research has shown that typical laboratory rat cages do not provide sufficient vertical space [11,133], but lab animal stakeholders interviewed about cage height were generally unreceptive to change, citing a lack of scientific evidence [134]. In this example, stakeholders were seemingly influenced by their own experiences, assumptions about rat behaviour, or a desire to generate their own data. A similar phenomenon has been documented regarding the refinement of mouse handling methods; research on the effects of tail-handling has been conducted in several laboratories with different strains of mice [135,136], but respondents in a recent survey expressed a desire for more evidence [101].

To aid progress, policy makers may wish to establish clear guidelines of the type and quantity of evidence required before adopting refinements, as well as who is expected to generate and evaluate this data. We encourage caging manufacturers to assess products for demonstrable animal welfare benefits before putting them on the market. It may also be useful to define what scientific criteria are necessary for researchers to opt out of providing recommended enrichment.

#### 3.3.7. Risks to Animal Health or Safety

Although a major aim of enrichment is to improve the welfare of animals, the misapplication or misidentification of enrichment can have the opposite effect. Negative effects on animal health, behaviour, and wellbeing were identified as risks of enrichment in several articles. Some of these articles identified how poor choices of enrichment materials could increase injury or disease [7,72,74]. For example, paper tissues have been identified as inappropriate enrichment for mice used in asthma studies, because the cellulose fibres of these tissues contribute to inflammatory reactions of the lungs [137]. In another case, fibrous nesting material was noted as causing injury to mice [138]. Therefore, even for widely endorsed forms of enrichment such as nesting material, some options will be better than others and approaches may need to be altered depending on context. Several articles recommended certain enrichment strategies only conditionally, often citing the potential for increased aggression among male mice [37,54,58,59,64,67].

Aggression among male laboratory mice is a complex issue. Aggression is a natural component of rodent behaviour and has rewarding properties [139], so providing mice with the freedom to express some of this behaviour (so long as subordinates can escape without harm) could be considered to provide welfare benefits. It is possible that previous studies have failed to provide sufficient quantities of enrichment to mitigate competition [140]. While there are many examples of structural enrichment resulting in increased aggression among males (e.g., [141,142]), there are also many cases of successful enrichment use (involving a range of genetic strains and enrichment strategies) [143,144,145,146,147]. In one example, male mice housed with hemp ropes hanging from the cage lid demonstrated more aggression when frequently tail-handled by experimenters; when handling was reduced, mice housed with hemp ropes showed no differences in aggression compared to the conventionally housed control group [145]. Therefore, reactions to enrichment could be context specific. We recommend further research into the welfare of male mice housed in more complex environments, specifically relating to causes and mitigation strategies for excessive aggression.

#### 3.3.8. Other Risks and Requirements

The articles we reviewed also occasionally cited other risks or requirements of enrichment: potential for conflicts with standardised practices or ongoing experimental protocols (8 articles); staff must be motivated and knowledgeable about rodent behaviour (8 articles); issues related to existing facilities or cage dimensions (7 articles); resistance from personnel or researchers (5 articles); enrichment should not risk staff safety (4 articles); and enrichment should not impede visual inspection of animals (3 articles). One article specified that enrichment should be commercially available, while another suggested that enrichment must meet hygienic or biosafety requirements. Key [58] suggested that suitable enrichment must fulfil the “three Ps”: Proven (enrichment should result in increase in species specific behaviours, decrease stereotypies, and no unacceptable increase in variability), Practical (enrichment should be easy to use), and Price (enrichment should be inexpensive). Notably, two of these three components are human-related, as were most of the themes in the risks or requirements category. These statements also often reflected the authors’ own pragmatic concerns or the perceived concerns of other relevant stakeholders rather than representative data.

### 3.4. Reviewing and Implementing Enrichment

Some authors suggested how different factors should be weighed when considering the perceived risks described above. Baumans and Van Loo [69] suggested that animal-related factors, scientific validity of the animal model, and factors related to the animal facility must be equally addressed in relation to enrichment; Conour et al. [67] suggested that animal welfare must be balanced with sound scientific practices; Sørenson et al. [61] and Bayne and Würbel [70] suggested that an enrichment cost–benefit analysis should be conducted in which welfare benefits are weighed against potential harms to research.

Some articles indicated who is responsible for assessing and implementing enrichment, but it is unclear how these recommendations line up with the perceived responsibilities of stakeholders. The primary investigator and husbandry staff were often cited as being responsible for enrichment, while some also listed the facility veterinarian and the animal ethics committee [7,64,67,72]. In reality, lab animal husbandry personnel often report having little to no control over provision of enrichment [99]. Additionally, 95% of lab animal veterinarians surveyed in a recent study rated mouse welfare as acceptable to excellent in current conditions [148], so motivation to advocate for housing refinements may be low. In another study, most lab animal veterinarians and technicians agreed that institutional rules or regulations were sufficient to ensure quality of life for animals, and indicated that it was the responsibility of the animal ethics committee to address animal welfare concerns [149].

Although animal ethics committees play a role in reviewing and updating enrichment programs [150,151,152], it is unclear if ethics committees consider implementing refinements outside of experimental protocols as part of their role. A survey of non-human primate facilities in the United States found that only 36% of staff perceived strong support for enrichment from their institutional animal ethics committee [153]. Instead, these participants felt that inspections from regulators or accreditation programs prompted enhancements to enrichment programs much more often than did ethics committee reviews, suggesting that institutional animal ethics committees are not effective in promoting enrichment. Several factors may impede the promotion of enrichment by ethics committees, such as a general misunderstanding of what Refinement means, belief that animal welfare is already high, or a focus on mitigating pain caused by procedures rather than on quality of life related to animal husbandry [154]. Varied perceptions of individual responsibility for Refinement among lab animal stakeholders may represent a more implicit barrier to enrichment.

Decisions about what refinements to adopt will require some type of assessment and balancing of the benefits and risks. The discerning reader will have noted that there is extensive scientific research on the effects of environmental enrichment, but risks and requirements are sometimes stated without strong supporting evidence. We suggest that decision making processes place equal emphasis on the quality of evidence for both benefits and risks. Decision makers should also be aware of potential biases. For example, any cost–benefit analysis is likely to be subject to status quo bias (i.e., a preference to maintain the current state of affairs) as well as loss aversion (i.e., perceived risks or losses are viewed as more costly than bypassed gains; [155]). Thus, without appropriate safeguards in place, cost–benefit analyses of enrichment are likely to unduly favour the status quo, overestimate costs, and underestimate benefits. We call for decision making processes that explicitly articulate how risks and benefits were estimated and how decision makers attempted to avoid these biases.

### 3.5. Future Directions

There is considerable variation in the focus of contemporary animal welfare enrichment research. Research continues on the effects of more widely used housing additions such as shelters (e.g., [156,157]) and nesting material (e.g., [42,158]). Of note, studying the effects of a singular enrichment item is informative, but from a practical perspective this approach has been shown to be less effective at improving welfare than the provision of more extensive or diverse enrichment [111,159,160]. Alternative forms of enrichment such as positive reinforcement training [161] and burrowing (i.e., digging in a deep substrate to form burrows) [11,12] received less attention in the articles we reviewed; we encourage further research in these areas to better understand how to provide diverse behavioural opportunities that can benefit laboratory rodents.

While some aspects of laboratory housing could be altered immediately, it is likely not possible to provide ideal rodent environments within existing standard cages due to their restrictive dimensions [32,162]. Conditions that might allow for a “good life” in laboratory environments have been outlined by Makowska and Weary [32], but we may need considerably different housing conditions to achieve this [66]. Cage manufacturers have a key role to play in providing cages with dimensions that can accommodate more complex environmental components. In the more immediate future, creative enrichment solutions that overcome or work within current practical limitations are called for. Some examples of more immediately feasible enrichment include altered food provision to allow for more natural foraging [52], the provision of get-away tunnels or lofts to allow for more natural maternal care when dams are housed with pups [163,164], combining existing cages to allow for more space and structural components in a housing system [165,166], repurposing of cages meant for larger species [167], repurposing of existing facility materials to make enrichment components [143], promoting positive human–animal interactions [168,169,170], and the use of temporary playpens in facilities that lack the space or resources to permanently house animals in more complex environments [3,43,171]. Notably, the environments of laboratory animals that are not used in experiments (e.g., breeding, training, or sentinel animals) could be improved with fewer constraints, given that they are not bound to experimental requirements [73]. Almost half of all animal research procedures in the UK in 2019 involved breeding for the creation or maintenance of genetically altered animals [172]; this represents a considerable opportunity for animals to benefit from increased environmental complexity.

## 4. Conclusions

Environmental enrichment is most often conceptualised as a method to increase natural behaviour and improve animal welfare. We advocate for the adoption of specific and value-neutral descriptors to explain exactly what elements of the environment are modified and how they are believed to affect the welfare of the animals. Such descriptions would help to clarify that not every cage alteration is beneficial for welfare, and to avoid framing basic features of housing systems as luxuries. Many review articles supported providing rodents with social housing, nesting material, provision of shelters or nest boxes, opportunities for foraging, and (for rats at least) larger environments. Given the near ubiquity of these recommendations, we suggest that these be framed as basic housing components for laboratory rodents; regardless, we suggest that researchers justify the framing they have employed when describing housing components, and understand the effect of this framing on their conclusions. The papers we reviewed often described perceived risks or requirements of enrichment, such as the need for more empirical evidence, practical and financial constraints, and the potential for enrichment to alter variability of research outcomes. The quality of evidence for these concerns was often not clear; we suggest that decision makers take this into consideration when attempting to conduct a cost–benefit analysis for the provision of environmental enrichment.

## Figures and Tables

**Table 1:** Summary of definitions, goals, requirements or risks of environmental enrichment (EE), and recommended forms of EE, as described in review articles on the topic. “Additional requirements or risks of EE” consisted of any aspect that was indicated by authors, using words like “must” or “should”, as a necessity for successful implementation of enrichment, or otherwise identified as a barrier or potential risk of enrichment. “Recommended EE” lists components specified by authors as something that should (or should not) be provided to mice or rats; general discussions about forms of enrichment without a specific conclusion or recommendation were not included here. Note that definitions of EE often quoted or referenced other publications; these citations are excluded from the table.

Author and Year	Species	Definition of EE	Specific Goals of EE	Additional Requirements or Risks of EE	Recommended EE
Scharmann, 1991 [13]	Both	- Housing animals “in a manner conducive to their ‘psychological well-being’”	- Enable animals to express species-specific behaviours - Create circumstances “that will enhance the animals’ welfare, even to the point of... giving them the benefit of the doubt, or of being generous toward them”	- EE should not result in extensive additional work for staff - EE must meet hygienic requirements - EE should not impede inspection of the animals - Resistance from experimenters	For rats: - Higher cages to allow for standing and sufficient space for play - Opportunities for activity such as gnawing or tugging paper material through the cage lid For mice: - Use of vertical cage space to provide opportunities for mice to climb - Nesting material - Foraging opportunities
Dean, 1999 [55]	Both	- “…any measure which promotes expression of natural, species-specific behaviours and a decrease in, if not disappearance of, abnormal behaviours”	- Improve animal well-being - Make animal lives more comfortable and interesting - Encourage “normal” behaviours, decrease abnormal behaviours	- The notion of “historical data” and the desire for results to be comparable to those of previous studies - The impact of confounding variables introduced by EE must be minimised - Resistance from researchers - Financial costs - Limited staff time	For rats: - Inclusion of solid inserts on grid floors, or the inclusion of a shelf within the cage - Hard pelleted diet - Background music to dull impacts of sudden noise For mice: - Plastic bottles or shelters - Pelleted diet For both: - Nest boxes with paper nesting material - Social housing
Galef, 1999 [56]	Both	- “…changes in the physical or social environment [to] increase rodents’ psychological welfare”	- Increase natural behaviour (absence of abnormal behaviours, maintenance of species-typical repertoire) - Maintenance of tame, docile (not fearful) behaviour - Increased psychological well-being of animals - Improved health (disease resistance, increased longevity and reproduction)	- Need precise specification of what constitutes failure or success of EE - Benefits of EE must be empirically proven - EE should not decrease animals’ suitability for laboratory life	- Recommends against increased cage sizes
Olsson and Dahlborn, 2002 [37]	Mice	- “… the practice of modifying housing conditions in order to promote natural behaviour and ameliorate behavioural problems” - “The term in itself implies an improvement beyond the satisfaction of basic needs”	- Improve animal welfare - Improved biological functioning - Increase natural behaviour - Decrease abnormal behaviour - Increase animal’s ability to cope with stressors - Maximise use of the environment	- EE should be systematically evaluated for animal welfare outcomes and effects on parameters relevant to experimental outcomes - Preference studies need to be combined with studies of motivational strength in order to draw animal welfare conclusions - Effects of EE may vary by strain or sex	- Nesting material - Shelter structures (conditionally) - Access to running wheels and larger or more structured cages (conditionally)
Johnson and Patterson-Kane, 2003 [52]	Rats	- “…a means of improving welfare”	- Improve welfare - Correct behavioural problems such as stereotypies or apathy	- EE must be practical in terms of costs and labour - EE must be species appropriate - There may be experimental constraints such as sterility of the environment or needing to monitor feed intake - Laboratory cages are restrictive in size	Foraging EE: - Small food particles mixed in with bedding - Mixing food in a dish with other substrates - Giving access to whole food pellets or variable food types - Addition of a shelf in the cage
Hawkins and Jennings, 2004 [57]	Both	- “[EE] for rodents is a positive way to improve their welfare” - "Providing good quality and quantity of space”	- Improve welfare	- Concerns about wasting time and money on items that the animals do not really need - Concerns that EE may increase variability in results - Lack of awareness about EE that has been successfully used and validated - EE should benefit animals without compromising scientific outcomes - Risk of cluttered cages that obstructs view of animals - Financial constraints - All staff should be knowledgeable and able to interpret rodent behaviour	- Social housing - Enough space for exercise and provision of EE - Enough cage height to rear - Solid floors - Adequate depth of appropriate substrate - Gnawing object - Shelter/nest boxes - Nesting material - Appropriate light levels - Foraging opportunities
Key, 2004 [58]	Both	- “[EE] is the alteration of animals’ microenvironments to provide them with the opportunity to perform species-specific behaviours that we perceive as positive, while reducing abnormal behaviours”	- Improve animal welfare - Increase species-typical behaviours - Decrease stereotypic behaviours	- EE should be proven effective by statistically significant increase in positive behaviours, together with reduction in abnormal behaviours - Risk of decreasing usable cage space - EE should not cause unacceptable increases in variability - EE should be practical to use - EE should be inexpensive	For both: - Group-housing of rats and female mice (male mice conditionally) - Nesting material - Nest boxes - Foraging opportunities For rats: - Larger and more complex cages for groups of rats For mice: - Plastic huts are recommended over wooden or disposable shelters
Ottesen et al., 2004 [59]	Both	- No definition provided	- Reduce stress-induced behaviours - Allow for species-typical behavioural patterns - Improve welfare - Give animals a degree of control or choice	- EE should be regularly reviewed and updated - EE must be appropriate for species-specific and individual animal needs - EE requires knowledge and commitment from staff - Implementing new EE often requires scientific evidence	For rats: - Social housing - Structured environment with increased space, especially vertical space for rearing - Gnawing and digging opportunities For mice: - Social housing (conditionally) - Nesting material - Access to darkness - Foraging opportunities
Patterson-Kane, 2004 [60]	Rats	- “It has been thoroughly demonstrated that barren housing conditions impair rats’ physical and behavioural systems, for example, by having effects on brain morphology, levels of fear, and performance on cognitive tests… “[EE]” research attempts to mitigate this damage and to improve animal welfare by modifying laboratory caging”	- Improve animal welfare	- Limited resources (e.g., staff time, financial costs) - Introduction of confounding variables and potential for increased variability - Risks to animal health - EE should have empirically proven benefits - EE needs to be tailored to meet animal needs as well as the requirements of the research - EE should not negatively impact research goals or economic viability of research - Biases of personnel may influence EE use - EE should be commercially available	- Social housing (groups larger than 2) - Larger cages - Solid, opaque shelters in the form of nest boxes rather than tunnels - Comfortable bedding and paper nesting material
Sørensen et al., 2004 [61]	Rats	- “Enhancing the complexity of the environments of captive animals is often referred to as [EE], and aims to have positive effects on the animals’ well-being”	- Increase welfare - Allow animals to perform a range of species-specific behaviours	- EE may cause increased variability - Cost–benefit analysis should include welfare benefits of EE vs. harms caused by the experimental procedures involved - EE can make standardisation difficult - EE may increase aggression - EE should be strain, age, and size appropriate	- Social companions - Variable cage heights - Shelters - Soft bedding
Van de Weerd et al., 2004 [53]	Both	- “[EE] strategies, which aim to improve the housing conditions of laboratory animals, are viewed as refinement”	- Enhance animal welfare	- EE may increase variation and number of animals used - Effects of EE should not be generalised - EE should be species, strain, and sex appropriate	- Nesting material - Shelters (conditionally) - Social housing; for male mice, housing in groups of 3 with provision of nesting material
Baumans, 2005 [62]	Both	- “...any modification in the environment of captive animals that seeks to enhance its physical and psychological well-being by providing stimuli meeting the animal’s species-specific needs” - “[EE] applies to heterogenous methods of improving animal welfare and includes everything from social companionship to toys”	- Give the animal a greater choice of activity and some control over its environment - Increase behavioural diversity - Reduce abnormal behaviour - Increase positive use of environment - Increase coping abilities	- EE should pose no risk to animals or humans - EE should not cause undesirable effects on experiments - EE should not increase the number of animals used - EE should be scientifically tested prior to use - Staff must be motivated, educated, and empowered to implement EE - EE must be described sufficiently in publications - Economic and practical considerations	For both: - Social housing - Structural complexity in the cage (e.g., shelter or cage divider) allowing for a level of environmental control - Nesting material For rats: - Nest box - Opportunity to dig and gnaw - Foraging opportunities
Bayne, 2005 [50]	Both	- “[EE] is generally considered to imply an increase in the complexity of the environment in which the animal lives, with the goal of enhancing the animal’s welfare” - “…can encompass the variety of food items offered to the animal; whether or not the animal is housed in a bedded cage (i.e., rodents); and additional “structural” en-hancements such as nest-building mate-rials, shelves/perches, hiding areas, ma-nipulanda (toys), exercise wheels, climbing/swinging apparatuses, water features, access to the outdoors, and much more”	- Enhance animal welfare	- Safety of the animal and the staff should be considered - EE must have a demonstrable beneficial effect on the animal - Effects of EE may impact experiments or introduce variables to experiments	- No specific recommendations
Benefiel et al., 2005 [63]	Both	- “…an increase in the complexity or naturalness of an enclosure with the goal of improving animal welfare”	- Improve animal health, fitness, or reproduction - Improve animal welfare	- Financial cost - EE should not compromise research outcomes - EE should not compromise animal health or well-being - Animal preferences should not be used as the basis for EE decisions - Potential for increased variability in research outcomes	- No specific recommendations
Hutchinson et al., 2005 [64]	Both	- “A method to improve quality of life” - “In addition to social activities, [EE] can be achieved by allowing and promoting physical exercise, foraging, manipulative and cognitive activities, as relevant to the species concerned”	- Provide animals with opportunities to express species-typical behaviours - Enhance physical and mental health	- Risk of impacting experimental design or outcomes - EE may have different impacts depending on species, strain, and age - Practical considerations (ease of use, safety) - EE must be affordable - Animal preference data should be linked with other measures of well-being to draw conclusions about EE - Animal care staff must be knowledgeable about natural behaviour of the species	For both: - Social housing For rats: - Structural enrichment (conditionally) For mice: - Nesting material - Recommend against “superenrichment”
Smith and Corrow, 2005 [65]	Both	- EE is often defined as a “change to the environment” - “[EE] is increas-ingly appreciated as a way to im-prove the well-being of rodents, providing them with oppor-tunities for species-specific behaviours that might be available to them in the wild” - “[EE] can be as simple as adding a tissue or a particular type of bedding material to the cage, or as complex as adding devices such as shelters, running wheels, blocks for chewing, or plastic tubes”	- Improve the health and welfare of animals - Increase the frequency and diversity of positive natural behaviours - Decrease the occurrence of abnormal behaviour - Maximise utilisation of the environment - Increase the animal’s ability to cope with the challenges of captivity	- Potential for increase in experimental variability; EE should not cause significantly more animals to be needed - EE changes should not affect the dimensions of the caging systems currently in use - EE should be cost-effective - EE must be strain and sex appropriate	For rats: - social housing For mice: - nesting material
Balcombe, 2006 [66]	Both	- No definition provided	- Allow for normal or motivated behaviours	- Practical challenges in changing existing housing systems - Financial costs	- Social housing for mice and rats; aggressive male mice may benefit from creative husbandry solutions rather than isolation - Increased space - Nesting material - Shelter
Conour et al., 2006 [67]	Both	- “[EE] is a combination of complex inanimate and social stimulation”	- Maximise species-specific behaviours - Minimise stress-induced behaviours	- Potential for varied effects of EE depending on age, sex, and strain - Risk of introducing variables that impact research outcomes - EE should be biologically relevant - Potential for aggression caused by EE	Recommended conditionally: - Social housing - Nesting materials - Gnawing materials - Bedding - Shelters
Würbel and Garner, 2007 [54]	Mice	- “We distinguish between [EE] as an experimental variable (meaning adding inanimate and/or social stimuli to the environment) and its consequences in terms of animal welfare, and use the term beneficial enrichment for cases where [EE] results in improved animal welfare”	- Improve animal welfare	- Success of EE may depend on sophistication of management practices - EE should be biologically relevant and have low or no risk of negative welfare consequences - Risk of resistance from scientists aiming for environmental standardisation	- Nesting material - Shelters (conditionally) - Recommend against pseudoenrichment (e.g., marbles)
Brown, 2009 [51]	Both	- “In addition to social activities, [EE] can be achieved by allowing and promoting physical exercise, foraging and manipulative and cognitive activities”	- Promote natural behaviours- reduce stereotypies	- Dietary or other restrictions of scientific studies - Financial concerns - EE needs to be appropriate for the specific experimental circumstances	Foraging EE: - hay cubes, fruit or vegetable-based treats, and diets consisting of seeds and grains that can be delivered to the animals in a variety of ways, such as within a dry pasta shell or wooden toy with holes
Simpson and Kelly, 2011 [68]	Rats	- “[EE] is a term for exposing laboratory animals to physical and/or social stimulation that is greater than they would receive under standard housing conditions”	- Reduce stereotypies - Improve welfare	- EE should be biologically relevant - EE should be appropriately validated - EE must not increase variability or increase the number of animals required in studies - EE should be age, strain, and sex appropriate	- A combination of both social and physical enrichment elements is recommended - Larger cages - Social housing
Toth et al., 2011 [7]	Both	- “EE has been defined as the use of housing conditions that offer enhanced sensory, motor, and cognitive stimulation of brain neuronal systems in comparison with standard caging and, alternatively, as adding biologically relevant features to the cage environment to facilitate or allow the performance of natural motivated behaviors. Although these definitions are not mutually exclusive, the perspectives and probably the goals are clearly different... EE can take many forms”	- Stimulation of positive species-typical behaviours and/or prevention of abnormal or undesirable behaviours - Promote well-being	- EE must not confound experimental outcomes and/or should alleviate harm that occurs in the absence of the EE - Risk of disease - Personnel time and safety - Conflicts between refinement and reduction if animal numbers increase as a result of variability - EE should not endanger animals or reduce well-being - EE should either improve or leave experimental results unaltered - EE should not jeopardise experimental design - EE should be designed, assessed, and implemented based on judgment of IACUCs, husbandry personnel, and research staff - EE should be practical - EE should be supported by scientific data	- Recommend evaluation of EE on a case-by-case basis
Baumans and Van Loo, 2013 [69]	Both	- “[EE] can be defined as any modification in the environment of captive animals that seeks to enhance its physical and psychological well-being by providing stimuli which meet the animals’ species-specific needs”	- Provide stimuli beyond satisfaction of basic needs - Benefit animal well-being and experimental outcomes	- EE should be practical - EE should meet animals’ needs - EE should be inexpensive - EE should pose no risk to humans, animals, or the experiment - EE should be empirically supported by research - Factors important to the animal, scientific validity of the animal model, and the animal facility must be equally addressed - Staff must be motivated and educated - Potential for impacts on scientific outcomes or statistical power	- Nesting material - Chewable items - Opportunities for foraging - Social contact
Bayne and Würbel, 2014 [70]	Both	- “…[EE] has been described as a means to increase the amount of time an animal spends in species-typical activities (e.g., foraging, nest building), with a concomitant reduction in time spent expressing abnormal behaviour such as stereotypic locomotion and self-injurious behaviour” - “…inappropriate enrichment can induce fear or stress in an animal, and thus it is most accurate to speak in terms of providing beneficial enrichments, which improve an animal’s welfare”	- Increase the amount of time an animal spends in species-typical activities - Improve animal welfare - Expand range of possible behaviours - Address or prevent abnormal behaviour	- Must consider the safety of the animals and personnel - Physical and operational constraints of laboratory facilities - Staff must be knowledgeable - EE program should account for age, strain, and sex of the animals - EE should not preclude care staff from conducting daily husbandry duties - Need to understand potential ramifications of EE on the animal’s biology and whether this may have consequences for research outcomes	For both: - Social housing - Opportunities for physical and cognitive activity, such as foraging - Shelters For mice: - Nesting material
Jirkof, 2015 [71]	Mice	- “[EE] efforts in routine housing systems are, in terms of costs and practicability, less complex than cage enrichment in neu-robiology research. Often these efforts involve the addi-tion of biologically relevant features to the cage, creating a more natural set-ting, with the aim of facilitating or enabling the animals to engage in natural behaviours”	- Promote animal welfare - Facilitate natural behaviours - Enhance physical and emotional well-being	- EE should not increase variation or negatively impact experimental results - EE should not necessitate increased animal numbers - Effects of EE may be sex and strain dependent	- Familiar environments with stable social groups whenever possible (especially females) - Recommend evaluation of EE on a case-by-case basis
Bayne, 2018 [72]	Mice	- “… a method to enhance animal well-being by providing animals with sensory and motor stimulation, through structures and resources that facilitate the expression of species-typical behaviours and promote psychological well-being through physical exercise, manipulative activities and cognitive challenges according to species-specific characteristics”	- Reduce stereotypic behaviour - Increase expression of species-typical behaviours - Improve animal welfare	- EE should be implemented with input from the investigator, the veterinarian, and husbandry staff - EE must be thoroughly researched and evidence-based - EE should not negatively impact health or safety of animals - EE must be biologically relevant	- Nesting material - Nest boxes/shelter
Lewejohann et al., 2020 [73]	Mice	- “…improving housing conditions”	- Enhance animal welfare - Provide opportunities for species-typical behaviour - Provide opportunities to engage in rewarding behaviours - Reduce boredom	- Financial costs - Need for qualified personnel and available space - Possible interferences with experimental design or increase in variability of results - Possible sex differences in how EE affects the animals	- Nesting material - Burrowing opportunities - Gnawing substrate - More space to engage in locomotory play behaviour - Cognitive training
Pritchett-Corning, 2020 [74]	Both	- Used the term en-vironmental com-plexity (EC) - “[EC] is, by necessity, defined by comparisons rather than by a specific description. Two boundaries could be posited: a barren environment and the environment as experienced by wild animals. In general, environmental complexity could be lumped into standard enrichment, super-enrichment, and semi-naturalistic environments”	- Provide animals with opportunities to exhibit natural behaviours or meet highly motivated needs - Reduce animal stress by allowing them to gather information or have control	- Assessment of EC should be rigorous and may need to be repeated due to strain and sex interactions - EC should have some relation to a species’ natural environment - Practical problems, such as lack of space for larger cages or sourcing and sanitising of EC objects - Potential for injury to animals - Increased labour required to maintain - Potential to disrupt ongoing research	- “Standard” EE: cage with bedding, nesting material, shelter (conditionally), and a social partner - “Superenriched” environment should offer greater variety of objects, more space, more social partners - “Semi-naturalistic” enclosures should offer more opportunities for natural behaviours; may allow outdoor access, orders of magnitude larger than other housing options - Further efforts toward working with rodents in semi-naturalistic settings should be pursued
Kentner et al., 2021 [46]	Both	- “[EE] is one form of complexity that includes physical, sensory, cognitive, and/or social stimulation which provides an enhanced living experience to laboratory animals, relative to standard housing conditions”	- Promote natural behaviours - Promote typical brain functioning - Provide an enhanced living experience to animals	- Availability of resources (financial, physical space) - Feasibility (e.g., personnel constraints) - Effects of EE may vary with species, age, or sex - Changing EE standards will require changes in mindsets of institutions, scientists, and funding bodies - Concerns about variability	- Social housing - Use larger cages that would take up the same space as several smaller cages to house larger groups - Regularly rotating EE for novelty

## Data Availability

Not applicable.
